# Supplementary material for: Complexome profiling of the Chlamydomonas psb28 mutant reveals TEF5 as an early PSII assembly factor
Source: Plant Cell. 2025 Mar 17;37(6):koaf055. doi: 10.1093/plcell/koaf055 (PMC12164590; doi:10.1093/plcell/koaf055)
Supplement: koaf055_Supplementary_Data [file koaf055_supplementary_data.zip › psb28_interactive.zip!/plots/intensity/qualityPlots/peptides/EFG8_Cre06.g259150.t1.2.html]

### Cre06.g259150.t1.2

Trivial name: EFG8  
SeqCoverage [median]: 22.50  
Mass: 48.02 kDa  
  
 

| Sequence | MissedCleavage | Mass [Da] | wtIntensity | muIntensity | Score |
| --- | --- | --- | --- | --- | --- |
| AGEDIEIVGLKDTIK | 1 | 1599.87 | 375.55 | 0.00 | 34.43 |
| LMQAVDDYVLVPER | 0 | 1646.83 | 0.00 | 1636.32 | 145.31 |
| SLGQGQAGDNVGLLVR | 0 | 1582.84 | 0.00 | 2031.99 | 154.36 |
| TKPHLNVGTIGHVDHGK | 1 | 1808.96 | 1882.08 | 0.00 | 30.57 |
| VVAYDQIDKAPEEK | 1 | 1603.81 | 0.00 | 1084.85 | 42.21 |
|  |  |  | 2257.6 | 4753.2 |  |
